# Concentration of Secreted Frizzled-Related Proteins (SFRPs) in Non-Small Cell Lung Carcinoma Subtypes—A Preliminary Study

**DOI:** 10.3390/curroncol30110724

**Published:** 2023-11-18

**Authors:** Jadwiga Gaździcka, Agata Świętek, Dorota Hudy, Natalia Dąbrowska, Karolina Gołąbek, Mateusz Rydel, Damian Czyżewski, Joanna Katarzyna Strzelczyk

**Affiliations:** 1 Department of Medical and Molecular Biology, Faculty of Medical Sciences in Zabrze, Medical University of Silesia in Katowice, 19 Jordana St., 41-808 Zabrze, Poland; 2 Silesia LabMed Research and Implementation Center, Medical University of Silesia in Katowice, 19 Jordana St., 41-808 Zabrze, Poland; 3 Strathclyde Institute of Pharmacy and Biomedical Sciences, University of Strathclyde, 161 Cathedral Street, Glasgow G4 0RE, UK; 4 Department of Thoracic Surgery, Faculty of Medical Sciences in Zabrze, Medical University of Silesia in Katowice, 13/15 3-Go Maja St., 41-800 Zabrze, Poland

**Keywords:** non-small cell lung cancer (NSCLC), secreted frizzled-related protein (SFRP), SFRP1, SFRP2, SFRP5, ELISA, tumour, protein concentration

## Abstract

Non-small cell lung carcinoma (NSCLC) is the most common lung cancer worldwide. Secreted frizzled-related proteins (SFRPs) are important tumour suppressors and antagonists of the Wnt signalling pathway, which is linked with cancer development. The aim of this study was to evaluate the concentrations of SFRP1, SFRP2, and SFRP5 proteins in tumour and non-tumour (NT) samples obtained from 65 patients with primary NSCLC. An enzyme-linked immunosorbent assay (ELISA) was used to measure the concentrations of SFRPs in the tissue homogenates. A significantly lower SFRP2 protein concentration was found in the total NSCLC tumour samples and the following NSCLC subtypes: squamous cell carcinoma (SCC) and adenocarcinoma (AC) (*p* > 0.05, *p* = 0.028 and *p* = 0.001, respectively). AC tumour samples had a higher SFRP1 level than NT samples (*p* = 0.022), while the highest SFRP1 concentration was found in NSCLC samples from patients with clinical stage T4 cancer. Increased concentrations of SFRP1 and SFRP5 were present in stage III NSCLC samples, while the tumour samples with high pleural invasion (PL2) had an increased level of SFRP2. The results from this study suggest that the tumour suppressor or oncogenic roles of SFRPs could be connected with the NSCLC subtype. The levels of SFRPs varied according to the clinicopathological parameters of NSCLC.

## 1. Introduction

Lung cancer is the most common cancer, affecting 1.8 million people worldwide each year. It is one of the most aggressive malignancies, with a 5-year survival rate of about 22% [1,2,3,4]. The diagnosis is usually made at an advanced stage of the disease, when treatment options are limited. Therefore, worldwide mortality rates are similar to incidence rates [4,5]. In 2019, in Poland, lung cancer was the second most common cancer among men (16.1%) and women (9.9%) and was the leading cause of death in both sexes [6]. The number of new cases is expected to increase in the coming decades [7]. Cigarette smoking is a risk factor that plays a pivotal key role in lung cancer etiopathogenesis. Other risk factors include exposure to second-hand smoke, arsenic, metals, fibres and dust, organic compounds, and radioactive elements [1,8,9,10]. The negative effect of air pollutants is also crucial; PM10 and PM2.5, nitrogen and sulphur oxides, and ozone could increase the risk of lung cancer [11,12]. The classic histological classification of lung cancer includes two main groups—small cell lung carcinoma (SCLC) (15% of cases) and non-small cell lung carcinoma (NSCLC) (85% of cases) [3,13]. NSCLC is divided into three subtypes: adenocarcinoma (AC) (40%), squamous cell carcinoma (SCC) (25–30%), and large cell carcinoma (LCC) (5–10%) [8,14,15]. 

The Wnt signalling pathway participates in the regulation processes related to embryogenesis and cell proliferation and differentiation [16,17]. Glycoproteins belonging to the secreted frizzled-related protein (SFRP) family are important extracellular signalling ligands and antagonists of the Wnt pathway. The SFRP family includes five members (SFRP1-5) and inhibits the Wnt pathway by binding to frizzled (FZD) family receptors [18,19]. On the other hand, it is also possible to bind SFRPs (with a domain very similar to the extracellular Wnt binding domain of frizzled receptors) to Wnt ligands, blocking the interaction between the Wnt receptor and the frizzled receptor [20]. Any dysfunction of these pathways is associated with the onset of multiple pathologies, which can ultimately also lead to cancer [21]. 

SFRP1 is the most researched molecule of all the SFRP family proteins, and its tumour suppressor activity has been well established in various types of cancer, such as intrahepatic cholangiocarcinoma and cutaneous squamous cell carcinoma [22,23,24]. Additionally, a decreased concentration of SFRP1 has been described in some human cancers, suggesting that SFRP1 suppresses proliferation, migration, and invasion [25,26,27]. It is supposed that a decreased level of SFRP1 could result from both epigenetic and genetic mechanisms, including the processes of DNA methylation, non-coding RNA, genomic alterations, and allelic imbalance [28,29,30]. Interestingly, it has been proven that SFRP1 may have the ability to affect Wnt pathway signalling, depending on its concentration, respectively, acting at low concentrations to activate and high concentrations to inhibit [31]. SFRP2 is a protein which can play a dual role, as an antagonist and agonist of the WNT signalling pathway [32]. SFRP5 is an adipocytokine with anti-inflammatory properties and is well studied in obesity and related diseases [33,34]. Moreover, it is an important tumour suppressor [35].

Changes in the mRNA expression of SFRPs have been found in multiple cancers, including lung cancers, and have been associated with cancer risk and prediction [32]. In several malignancies, including cervical cancer, breast cancer, ovarian cancer, prostate cancer, and renal cancer, changes in the concentration levels of the SFRP family have been demonstrated [36,37,38,39,40]. The relationship between changes in the concentrations of SFRPs and subtypes of NSCLC is still unknown. To the best of our understanding, it remains unclear whether alterations in SFRP concentrations have any association with NSCLC. Therefore, it was decided to analyse SFRP concentrations in samples collected from NSCLC patients.

This is the first study to evaluate the concentrations of SFRP1, SFRP2, and SFRP5 in tumour and non-tumour (NT) samples from patients with primary NSCLC, including adenocarcinoma, squamous cell carcinoma, and large cell carcinoma. The possible associations of clinical and demographic variables with the concentration of the selected proteins in the Polish population were also analysed.

## 2. Materials and Methods

### 2.1. Patients and Samples

The study population comprised 65 patients recruited from the Department of Thoracic Surgery, Faculty of Medical Sciences, Zabrze, Medical University of Silesia in Katowice. Inclusion criteria were a diagnosis of NSCLC and consent to participate in the study. The exclusion criteria were a diagnosis of lung cancer other than non-small cell lung cancer and a lack of patient consent to participate in the study. All patients were diagnosed via computed tomography (CT)-guided lung aspiration biopsy. In total, 65 tumour and non-tumour (NT) samples were obtained during surgical resection. The tumour samples contained histopathologically confirmed NSCLC cells. The NT samples were taken from macroscopically unchanged tissue at >3 cm distance from the tumour, and the presence of cancerous cells was excluded by pathologists. All tumour and NT samples were immediately frozen at −75 °C until homogenisation. All patients underwent R0 resection and no STAS (spread through air spaces) were found. The tumour stage was evaluated according to the 8th edition of the TNM classification [41]. This study was approved by the bioethics committee of the Medical University of Silesia in Katowice, Poland (No. PCN/0022/KB1/72/I/20/21). The collected samples were transported on ice to the laboratory of the Department of Medical and Molecular Biology of the Faculty of Medical Sciences in Zabrze of the Medical University of Silesia in Katowice, where all analyses were conducted.

### 2.2. Homogenisation

The methodology for homogenisation was presented in a previous study [42]. The tumour and NT samples were homogenised in PBS (EURx, Gdansk, Poland) at a ratio of 9:1 (PBS volume/tissue weight). Homogenisation was conducted using a Bio-Gen PRO200 homogeniser (PRO Scientific Inc., Oxford, CT, USA) at a speed of 10,000 rpm. The resulting homogenates were then sonicated with an ultrasonifier: a UP100H (Hielscher, Teltow, Germany).

### 2.3. Determination of SFRP1, SFRP2, and SFRP5 Protein Concentrations

The methodology for protein concentration analysis was presented in a previous study [42]. An enzyme-linked immunosorbent assay (ELISA) was used to determine the concentrations of selected proteins in the homogenates. Commercially available ELISA kits were used for SFRP1, SFRP2, and SFRP5 proteins (respectively, SEF880Hu, SEF879Hu and SEC842Hu, from Cloud-Clone Corp., Houston, TX, USA). The analyses were performed according to the manufacturer’s instructions. The detectable dose sensitivity for SFRP1 was 0.057 ng/mL, 6.1 pg/mL for SFRP2, and 0.60 ng/mL for SFRP5. The intra-assay and inter-assay precisions for all kits were <10% and <12%, respectively. Absorbance reading of the samples was performed at 450 nm using a Synergy H1 microplate reader (BioTek, Winooski, VT, USA). In total, 100 µL of homogenates for SFRP1 and SFRP5 assays was used, and for SFRP2, 100 µL of 50× diluted homogenates was applied to the assay. Results were calculated in Gen5 2.06 software (BioTek, Winooski, VT, USA).

### 2.4. Total Protein Concentration Determinations

The methodology for protein concentration analysis was presented in a previous study [42]. The quantification of the total protein in the homogenates was carried out using the AccuOrange™ Protein Quantitation Kit (Biotium, Fremont, CA, USA) according to the manufacturer’s protocol. The assay had a detection range of 0.1–15 µg/mL. Samples were diluted 100-fold. The fluorescence measurements were analysed in a Synergy H1 microplate reader (BioTek, Winooski, VT, USA) at excitation and emission wavelengths of 480/598 nm, respectively. The concentrations of the analysed proteins for each sample were normalised with reference to the total amount of protein in the tissue lysates, and the value was expressed in pg/µg for all analysed SFRPs.

### 2.5. Statistical Analyses

Results were tested using the Shapiro–Wilk test to determine the normality of the data. Student’s *t*-test or the Mann–Whitney U test was used to verify the significance of differences in means or medians between groups. The correlation was determined with Spearman’s rank correlation coefficient. The level of significance was set at *p* < 0.05. Results are presented as mean ± SD or median with quartile range. Analyses were performed using Statistica 13.1 (TIBCO Software Inc., Palo Alto, CA, USA).

## 3. Results

### 3.1. Study Group

The study group consisted of 65 patients with confirmed non-small cell lung cancer. The mean age was 69.09 (±7.13) years. Forty-seven (72.30%) patients admitted to smoking tobacco products. The characteristics of the study group are presented in Table 1.

The absence of distant metastasis was determined using imaging techniques, such as computed tomography (CT) and positron emission tomography/image-based computed tomography (PET/CT). As a result, this group of patients was homogeneous in parameter M (a component in the TNM classification). In patients with enlarged mediastinal lymph nodes, found through imaging studies, endobronchial/endoscopic ultrasound (EBUS/EUS) was performed. The final pathology TNM (pTNM) classification was determined through the postoperative histopathological examination of resected tumour samples.

### 3.2. Concentration of the Selected SFRPs in NSCLC Subtypes 

Analysis of the total NSCLC group showed significantly lower SFRP2 protein concentration in tumour samples than in the NT tissue (189.45 vs. 614.22) (*p* < 0.001). No statistically significant differences were found in the protein levels of SFRP1 or SFRP5 between tumour and NT samples, but their median concentration was slightly higher in the tumour samples. The concentration levels of SFRPs are presented in Table 2.

Analysis according to NSCLC subtypes showed similar results, which are presented in Table 3. 

For the AC subtype, significantly higher concentrations of SFRP1 and SFRP5 in tumour samples compared to NT were detected (*p* = 0.022 and *p* = 0.011, respectively), while the level of SFRP2 protein was reduced in tumour samples (*p* < 0.001). 

For the SCC subtype, a lower SFRP2 concentration in tumour specimens than in NT was observed (*p* = 0.028). However, the levels of SFRP1 and SFRP5 were not significantly different between tumour and NT samples (*p* > 0.05). 

For the LCC subtype, no significant differences in the levels of any analysed SFRPs were observed between tumour and NT samples (*p* > 0.05).

**Figure 1:**
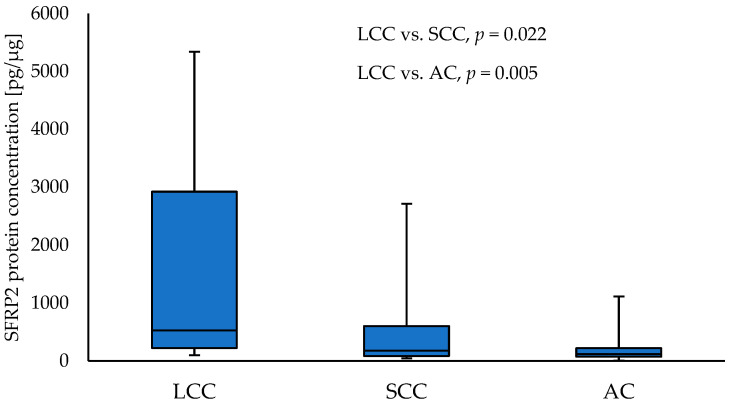
The SFRP2 protein concentration in the tumour samples according to NSCLC subtypes.

Comparisons between NSCLC subtypes showed a significantly higher level of SFRP2 in LCC tumour samples compared to SCC (528.85 vs. 178.18; *p* = 0.021) and AC (528.85 vs. 116.57; *p* = 0.004) tumour samples, respectively. The results are given in Figure 1. No other differences in SFRP concentrations between NSCLC subtypes’ tumour samples were reported.

**Figure 2:**
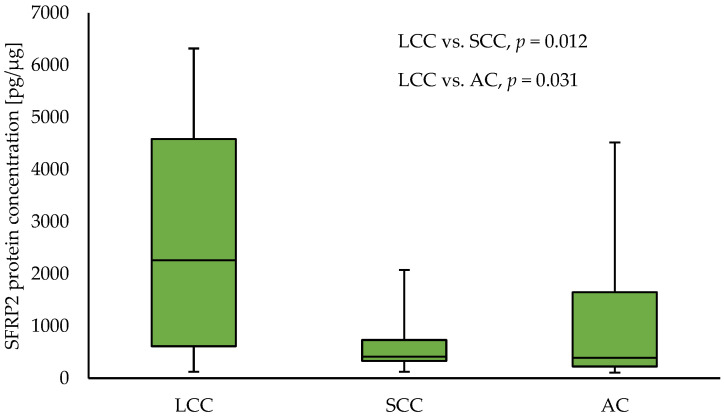
The SFRP2 protein concentration in the non-tumour samples according to NSCLC subtypes.

Similar to the results for the tumour samples, in NT, an increased level of SFRP2 in LCC samples compared to SCC (2258.59 vs. 414.07; *p* = 0.012) and AC (2258.59 vs. 391.96; *p* = 0.030) NT samples, respectively, was observed. The results are presented in Figure 2. In NT samples collected from SCC patients, higher levels of SFRP1 and SFRP5 protein compared to samples obtained from AC patients (SFRP1: 174.27 vs. 112.77; *p* = 0.0.28, and SFRP5: 5995.4 vs. 2512.5 *p* = 0.003), respectively, were observed. No other differences in levels of any analysed SFRPs between NSCLC subtypes for NT samples were found.

### 3.3. Protein Levels of SFRPs and Clinicopathological and Demographic Parameters

The number of samples in the NSCLC subtype groups were insufficient to perform analyses with clinicopathological parameters. Therefore, the results for the total NSCLC group are presented.

#### 3.3.1. Concentration of SFRP1 and Clinicopathological and Demographic Parameters

**Figure 3:**
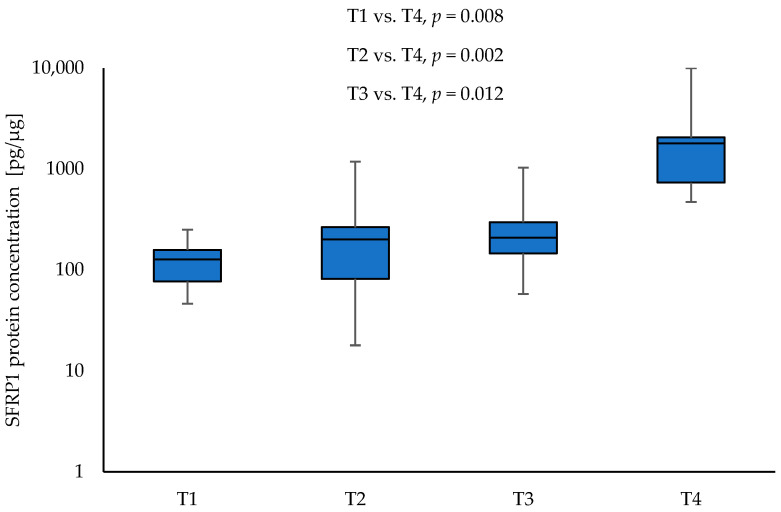
The SFRP1 protein concentration in the tumour samples according to T1, T2, T3, and T4 (logarithmic scale).

**Figure 4:**
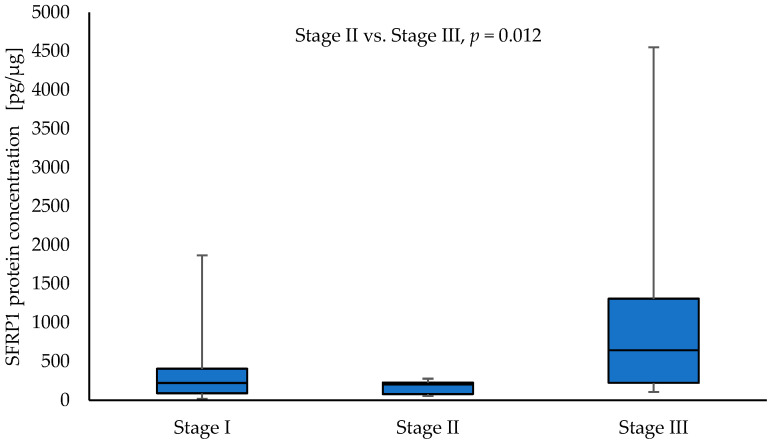
The SFRP1 protein concentration in the tumour samples according to stage I, stage II, and stage III in a group of NSCLC patients.

SFRP1 protein concentration was significantly higher in tumour samples from patients with clinical stage T4 compared to NSCLC samples with other T parameters: T1 vs. T4 (126.84 vs. 1790.35; *p* = 0.008), T2 vs. T4 (200.85 vs. 1790.35; *p* = 0.002), and T3 vs. T4 (208.83 vs. 1790.35; *p* = 0.012), respectively. Moreover, the level of SFRP1 was significantly higher in tumour samples obtained from patients with stage III NSCLC compared to tumour samples obtained from patients with stage II (644.83 vs. 204.38; *p* = 0.012). The results are given in Figure 3 and Figure 4. No significant association was found between the level of SFRP1 protein in tumour samples and other parameters (N, G, PL, sex, and smoking) (*p* > 0.05). 

#### 3.3.2. Concentration of SFRP2 and Clinicopathological and Demographic Parameters

**Figure 5:**
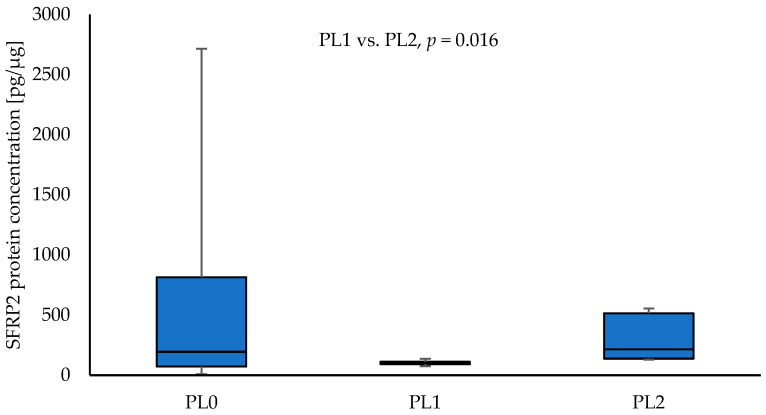
The SFRP2 protein concentration in the tumour samples according to PL0, PL1, and PL2.

Significantly higher SFRP2 protein levels were found in the tumour samples collected from patients with pleural invasion PL2 in comparison to the patients with PL1 NSCLC (214.96 vs. 103.05; *p* = 0.016) (Figure 5). The SFRP2 protein level was not significantly different between PL0 and PL1 (*p* = 0.233), as well as PL0 and PL2 (*p* = 0.640). No significant association between the level of SFRP2 protein and parameters, such as T, N, G, tumour stage, sex, and smoking, was observed (*p* > 0.05). 

#### 3.3.3. Concentration of SFRP5 and Clinicopathological and Demographic Parameters

**Figure 6:**
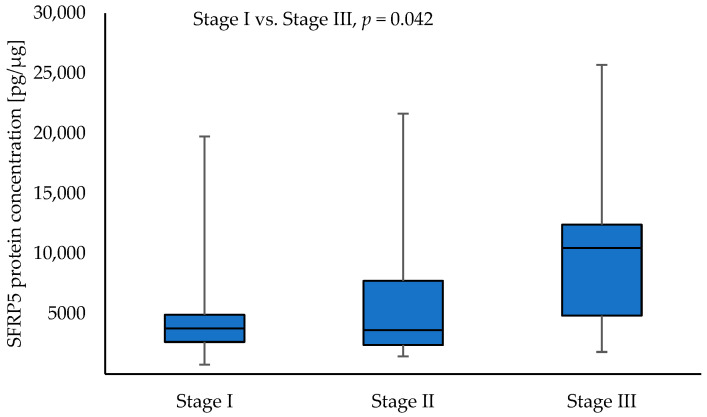
The SFRP5 protein concentration in the tumour samples according to stage I, stage II, and stage III.

Higher SFRP5 protein concentrations were observed in tumour samples with stage III compared to stage I NSCLC (10,475.35 vs. 3789.88; *p* = 0.042). The results are shown in Figure 6. 

No significant association between the concentration of SFRP5 protein and parameters (T, N, G, PL, sex, and smoking) was found in tumour samples. 

### 3.4. Correlations between SFRP Concentrations and Clinicopathological and Demographic Parameters

No statistically significant correlations between the concentrations of the selected proteins and any of the analysed clinical and demographic parameters were found. Moreover, the T parameter positively correlated with pleural invasion (*p* = 0.047; r_S_ = 0.269) and stage (*p* < 0.001; r_S_ = 0.791).

## 4. Discussion

The SFRP family is involved in regulation of the WNT signalling pathway, which is linked with cancer development [43]. The roles of the proteins SFRP1, SFRP2, and SFRP5 in the development of NSCLC are not fully understood. The main goal of this study was to analyse the protein concentrations of selected SFRPs among patients with subtypes of NSCLC, including AC, SCC, and LCC. Based on our knowledge, this is the first study that compares the concentrations of selected SFRPs among NSCLC subtypes. It has been observed that the SFRPs levels between tumour and non-tumour samples varied according to NSCLC subtype.

In this study, a significantly higher concentration of SFRP1 was found in the tumour samples compared to NT samples among patients with adenocarcinoma. On the other hand, the concentration of SFRP1 was insignificantly higher in SCC tumour samples and insignificantly lower in LCC tumour samples compared to NT samples. Moreover, higher concentrations of SFRP1 in NT samples collected from patients with SCC compared to AC was found. Importantly, in the literature, the level of SFRP1 was different for various cancers. A study based on breast cancer cell lines reported that SFRP1 protein level varied according to breast cancer subtype [44]. A decreased level of SFRP1 protein was observed in the head and neck squamous cell carcinoma [45], cutaneous squamous cell carcinoma [25], and gastric cancer [30], while increased protein concentration was reported in basaloid oesophageal squamous cell carcinoma samples [36] and breast cancer tissue specimens [44]. Interestingly, the bioinformatics analysis of mRNA *SFRP1* expression found significantly decreased *SFRP1* gene in tumour tissues compared to normal counterparts, in various cancers [32], suggesting that SFRP1 protein could possibly have different functions in different types of cancer [46]. The high expression of mRNA *SFRP1* has been associated with poor prognosis in lung squamous cell carcinoma and correlated with a better prognosis for patients with other types of cancer, including breast carcinoma, oesophageal adenocarcinoma, or head and neck squamous cell carcinoma [46]. Interestingly, Cheng et al. [46] revealed that high *SFRP1* mRNA levels correlated with different prognoses for patients with lung adenocarcinoma depending on the datasets used [46]. Moreover, we observed a significantly increased SFRP1 protein level in NSCLC samples with stage III and T4. Similar to these results, a Croatian study found that over 30% of renal cell carcinoma samples had higher SFRP1 protein levels compared to adjected normal tissues. An increased concentration of SFRP1 was observed in all samples obtained from patients with detected metastatic dissemination or higher Fuhrman grade, which assesses the aggressiveness of neoplastic cells [47]. Conversely, in mucoepidermoid carcinoma samples, a lower concentration of SFRP1 was associated with high-grade tumours [48]. It has been found that higher concentrations of SFRP1 reduce Wnt activity, while lower SFRP1 levels increase Wnt activity [31]. Therefore, it could be hypothesised that patients with AC would have a better prognosis than patients with LCC. Further studies are needed to determine the prognostic factor of SFRP1 levels for patients with various NSCLC subtypes. To our knowledge, no other reports with similar observations were in databases such as PubMed and Embase. 

SFRP2 is a tumour suppressor in various cancers and an important suppressor for NSCLC invasion [35,49,50]. In this study, a lower level of protein SFRP2 was observed in the total NSCLC group’s samples, as well as in SCC and AC tumours compared to non-tumour samples. We observed that the concentration of SFRP2 was the highest in LCC, compared to SCC and AC, in tumour samples, as well as in NT specimens. Similarly, some studies reported decreased SFRP2 protein levels in NSCLC specimens and NSCLC cell line A549 compared to non-tumour samples and pulmonary epithelial cell line BEAS-2B, respectively [49,51]. Moreover, the higher SFRP2 concentration decreased the survival, proliferation, and metastasis of NSCLC cells [51]. Therefore, it is suggested that SFRP2 could play a tumour suppressor role in NSCLC. However, SFRP2 could also be a tumour-promoting protein and an agonist to the Wnt pathway in lung cancer. A study based on three human lung cancer cell lines (95-D, SPCA-1, and A549) showed that the SFRP2 protein level was significantly higher in 95-D cells compared to A549 cells [52]. Similarly, we observed that the concentration of SFRP2 was the highest in LCC compared to SCC and AC, in tumour samples, as well as in NT specimens. Additionally, these authors analysed the effect of knockdown and overexpression of the SFRP2 protein and found that SFRP2 was an agonist of the WNT pathway and promoted the proliferation and invasion of lung cancer [52]. Therefore, it is hypothesised that the role of SFRP2 could be associated with the subtype of NSCLC. However, further studies on a larger cohort are needed to confirm this suggestion of the SFRP2 protein’s role in NSCLC subtypes. 

In this study, in patients with adenocarcinoma, a significantly higher concentration of SFRP5 was observed in tumour than in NT samples. Interestingly, it was observed that, in the total NSCLC group, an increased level of SFRP5 was present in tumour samples obtained from stage III NSCLC patients compared to stage I. In the literature, the SFRP5 protein concentration in cancers is still not explored enough. The antagonist role of SFRP5 in the canonical and non-canonical Wnt signalling path has been confirmed in breast cancer, where a higher level of *SFRP5* mRNA was associated with a better prognosis [53]. A reduction in SFRP5 was found in hepatocellular carcinoma and basaloid oesophageal squamous cell carcinoma [36,54]. Moreover, the mRNA analysis confirmed a lower expression of *SFRP5* gene in different primary tumours, including lung adenocarcinoma and lung squamous, compared to normal tissues [32]. Conversely, in patients with early-stage adenocarcinoma, the expression of the *SFRP5* gene was significantly higher in tumour samples than in normal tissues, and the authors suggested the oncogenic role of SFRP5 in lung adenocarcinoma [55]. Likewise, we observed a higher concentration in AC tumour samples compared to NT samples, and also could suggest the oncogenic role of SFRP5 in adenocarcinoma. However, the connections between the level of SFRP5 protein and tumour characteristics and prognosis for NSCLC patients has not been explored in the literature. Therefore, further studies on a larger cohort are needed to confirm the role of SFRP5 in NSCLC, especially in adenocarcinoma. 

SFRPs are not well studied regarding NSCLC; therefore, further studies are required to understand the connection between changes in SFRP concentrations and clinical and demographical parameters. The small sample size is the main limitation of this study. Our results should be validated on larger and more diverse cohorts. Moreover, further studies are needed to verify the impact of changes in SFRPs concentrations on the survival rate and prognosis in patients with NSCLC subtypes. 

## 5. Conclusions

In conclusion, this study found changes in selected SFRP concentrations in tumour samples and non-tumour samples, in patients with NSCLC, including adenocarcinoma, squamous cell carcinoma, and large cell carcinoma. These findings suggest that the tumour suppressor or oncogenic roles of SFRPs could be connected with the NSCLC subtype. In addition, we noted that SFRP levels varied according to clinicopathological parameters of NSCLC. Further studies are needed to verify how changes in SFRP concentration may affect tumour development and prognosis for patients.

## Figures and Tables

**Table 1:** Characteristic of the study group.

Parameter		n (%)
Sex	female	26 (40.00)
	male	39 (60.00)
Smoking	no (never)	8 (12.31)
	yes	47 (72.31)
	current smokers	31 (65.96)
	former smokers (in the past 12 month)	16 (34.04)
	NA *	10 (15.38)
T classification	T1	7 (10.77)
	T2	38 (58.46)
	T3	12 (18.46)
	T4	8 (12.31)
Nodal status (N)	N0	53 (81.54)
	N1	11 (16.92)
	N2	1 (1.54)
Stage	I	32 (49.23)
	II	20 (30.77)
	III	13 (20.00)
Histological grading (G)	G1	2 (3.07)
	G2	16 (24.62)
	G3	38 (58.46)
	NA *	9 (13.85)
Histological classification	Adenocarcinoma	28 (43.08)
	Squamous cell carcinoma	23 (35.38)
	Large cell carcinoma	14 (21.54)
Pleural invasion	PL0	37 (56.92)
	PL1	10 (15.38)
	PL2	8 (12.31)
	NA *	10 (15.38)

* NA—not assessed data.

**Table 2:** Concentrations of the selected SFRPs in tumour samples and non-tumour samples in the total NSCLC group.

Protein	Concentration in Samples [pg/µg] [Mediana (Lowest– Highest Quartile)]		
	Tumour	NT	*p*
SFRP1	214.83 (95.19–407.33)	126.78 (74.54–290.62)	0.057
SFRP2	189.45 (98.74–554.30)	614.22 (285.73–1953.11)	<0.001
SFRP5	4204.617 (2555.68–9470.96)	3744.23 (2377.14–6393.37)	0.225

**Table 3:** Concentrations of the selected SFRPs in tumour and NT samples in NSCLC subtypes: adenocarcinoma (AC), squamous cell carcinoma (SCC), and large cell carcinoma (LCC).

Protein	Concentration in Samples [pg/µg] [Mediana (Lowest–Highest Quartile)]								
	AC			SCC			LCC		
	Tumour	NT	*p*	Tumour	NT	*p*	Tumour	NT	*p*
SFRP1	180.07 (106.05–321.33)	112.77 (47.03–128.39)	0.022	220.85 (64.59–304.47)	174.27 (109.18–406.43)	0.552	208.83 (151.63–434.13)	247.44 (62.97–324.15)	0.555
SFRP2	116.57 (72.85–221.02)	391.96 (225.21–1647.72)	<0.001	178.18 (85.52–602.03)	414.07 (332.47–734.78)	0.028	528.85 (221.48–2922.49)	2258.59 (614.22–4582.93)	0.230
SFRP5	4129.36 (2994.06–9080.0)	2512.46 (1282.21–5212.65)	0.011	3871.61 (2503.91–5702.01)	5995.44 (3182.79–10,381.28)	0.142	6990.29 (2110.03–12,827.34)	4156.09 (2661.16–5114.55)	0.238

AC—Adenocarcinoma; SCC—Squamous cell carcinoma; LCC—Large cell carcinoma.

## Data Availability

The data presented in this study are available on request from the corresponding author.
